# Biological Atomic Force Microscopy for Imaging Gold-Labeled Liposomes on Human Coronary Artery Endothelial Cells

**DOI:** 10.1155/2013/875906

**Published:** 2013-02-21

**Authors:** Ana-María Zaske, Delia Danila, Michael C. Queen, Eva Golunski, Jodie L. Conyers

**Affiliations:** ^1^Division of Cardiology, Department of Internal Medicine, The University of Texas Health Science Center at Houston, 1881 East Road, Houston, TX 77054, USA; ^2^Division of Cardiology, Department of Internal Medicine, The University of Texas Health Science Center at Houston, 1841 East Road, Houston, TX 77054, USA; ^3^Center of Translational Injury Research, The University of Texas Health Science Center at Houston, 6410 Fannin Street, Houston, TX 77030, USA

## Abstract

Although atomic force microscopy (AFM) has been used extensively to characterize cell membrane structure and cellular processes such as endocytosis and exocytosis, the corrugated surface of the cell membrane hinders the visualization of extracellular entities, such as liposomes, that may interact with the cell. To overcome this barrier, we used 90 nm nanogold particles to label FITC liposomes and monitor their endocytosis on human coronary artery endothelial cells (HCAECs) *in vitro*. We were able to study the internalization process of gold-coupled liposomes on endothelial cells, by using AFM. We found that the gold-liposomes attached to the HCAEC cell membrane during the first 15–30 min of incubation, liposome cell internalization occurred from 30 to 60 min, and most of the gold-labeled liposomes had invaginated after 2 hr of incubation. Liposomal uptake took place most commonly at the periphery of the nuclear zone. Dynasore monohydrate, an inhibitor of endocytosis, obstructed the internalization of the gold-liposomes. This study showed the versatility of the AFM technique, combined with fluorescent microscopy, for investigating liposome uptake by endothelial cells. The 90 nm colloidal gold nanoparticles proved to be a noninvasive contrast agent that efficiently improves AFM imaging during the investigation of biological nanoprocesses.

## 1. Introduction

The development of biologically active nanoparticles that can facilitate the delivery of therapeutic and/or diagnostic agents to precise regions within the human body has gained widespread attention in the past 10 years. While many have successfully designed and tested such nanovectors *in vivo*, little is known about how these particles dynamically interact with cell membranes. Atomic force microscopy (AFM), a surface analytical technique that generates nanoscale topographical images under physiological-like conditions [1], combined with fluorescence microscopy is an attractive tool for characterizing nanoparticle-cell membrane interactions and may afford the opportunity to image biological processes, such as cellular endocytosis of nanovectors and reorganization of the cytoskeleton, in real time.

Liposomes were one of the first classes of engineered nanoparticles to be used to deliver drugs within the human body. One of the major disadvantages of using standard liposome formulations is their rapid clearance from circulation due to uptake by the reticuloendothelial system. To resolve this problem, long-circulating liposomes were developed in the early 1990s [2, 3]. Long-circulating liposomes have polyethylene glycol (PEG) derivatives attached to their surfaces. The conformational flexibility of PEG chains creates a sterical barrier that allows the liposomes to evade uptake by the reticuloendothelial system and remain in circulation for a longer time, increasing the possibility for targeting [4]. The ability to pegylate liposomes, for the purpose of mitigating a systemic immune response and/or enhancing prolonged vascular circulation, has made these particles attractive nanovectors for increasing the efficacy of therapeutics. For instance, Doxil entraps doxorubicin (an anthracycline antibiotic used as a chemotherapy agent) within a pegylated liposome for treating Kaposi's sarcoma [5]. 

Our laboratory is designing liposomes that are immunospecific to inflamed endothelium, a hallmark of atherosclerosis, for the purpose of imaging lipid-rich plaques before the onset of clinical symptoms (e.g., angina, coronary stenosis, etc.). While *in vivo* studies of liposomes plaque specificity (imaged by computed tomography) are currently underway, we are also determining how these liposomes interact with endothelial cells that have been induced to express an inflammatory response. 

Structure recognition can be a challenge during AFM imaging. Some nanoparticles can also be used as imaging contrast agents and as reinforcement elements to improve visual enhancement [6]. Colloidal gold has excellent detection capabilities for single-molecule tracking [7, 8]. The aim of this project was to standardize the use of gold nanoparticles as a nontoxic means to detect pegylated liposomes by AFM, on the membranes of live human coronary artery endothelial cells (HCAECs), to elucidate internalization processes. 

## 2. Experimental Methods

### 2.1. Materials

1,2-Dipalmitoyl-*sn*-glycero-3-phosphocholine (DPPC); cholesterol; 1,2-distearoyl-*sn*-glycero-3-phosphoethanolamine-N-[carboxy (polyethyleneglycol) 2000] (DSPE-PEG (2000)-COOH); and 1,2-distearoyl-*sn*-glycero-3-phosphoethanolamine-N-[methoxy (polyethyleneglycol)-2000] (DSPE-PEG (2000)) were purchased from Avanti Polar Lipids (Alabaster, AL, USA). N-(Fluorescein-5-thiocarbamoyl)-1,2-dihexa-decanoyl-*sn*-glycero-3-phosphoethanolamine (DHPE-FITC) was purchased from Molecular Probes (Eugene, OR, USA). Dimethyl sulfoxide anhydrous 99.9% (DMSO), N-(3-dimethylaminopropyl)-N′-ethylcarbodiimide hydrochloride (EDC), chloroform A.C.S. reagent 99.8% with amylenes as stabilizer, 2-(N-morpholino) ethanesulfonic acid (MES), collagen from calf skin, neutralbuffered formalin 10%, and dynasore monohydrate were purchased from Sigma-Aldrich (St. Louis, MO, USA). N-Hydroxysulfosuccinimide (sulfo-NHS) was purchased from Pierce (Rockford, IL, USA). Amine-PEG conjugated spherical gold nanoparticles (90-nm gold nanoparticles) were purchased from Nanopartz (Salt Lake City, UT, USA). HCAECs (CC-2585 Lot # EN000307), endothelial cell basal medium-2 (EBM-2), and the endothelial cell growth medium (EGM-2) BulletKit (SingleQuots) were purchased from Lonza (Walkersville, MD, USA). Eight-well Lab-Tek Chamber Permanox Slides were purchased from Nalge Nunc Int. (Rochester, NY, USA). Ruby red mica sheets (1 × 3′′), for liposome AFM imaging, were purchased from Electron Microscopy Sciences (Hatfield, PA, USA). AFM cantilevers for tapping mode (RTESP, *fo* = 262–325 kHz, *k* = 20–80 N/m) and contact mode (DNP-S, *fo* = 12–24 kHz, *k* = 0.06 N/m) were purchased from Bruker Corporation (formerly Veeco Metrology) (Santa Barbara, CA, USA).

### 2.2. Preparation of Long-Circulating Liposomes

We synthesized long-circulating liposomes by using DPPC and our previously published procedure [9]. The liposomes were composed of DPPC and cholesterol in a 3 : 1 molar ratio. The linker lipid DSPE-PEG (2000)-COOH was incorporated at a molar ratio of 3 : 1 : 0.3, DPPC : cholesterol : linker. The synthesized liposomes were sterically stabilized by addition of a 1% solution of DSPE-PEG (2000) in chloroform. For fluorescence microscopy experiments, 2 mol% of fluorescently labeled DHPE-FITC was incorporated into the lipid mixture. The liposomes were prepared by hydration of the dry lipid film as described by others [10, 11]. Briefly, the lipids were dissolved in chloroform, which was then carefully evaporated with a rotary evaporator (Büchi Corporation). The resulting dry lipid film was hydrated at 65°C with MES (100 mM, pH 5.5) to a final phospholipid concentration of 30 mM. Vigorous shaking of the solution produced large multilamellar vesicles of various sizes. Using an extruder (Avanti Polar Lipids), we passed the multilamellar vesicle solution 21 times through 0.2 *μ*m polycarbonate membranes at 65°C to yield a homogeneous solution of unilamellar vesicles with an average diameter of 129 nm, as determined by dynamic light scattering (DLS) measurements. 

### 2.3. Dynamic Light Scattering Measurements

DLS is one of the most common techniques to determine the radius of spherical particles in Brownian motion in a solution [12]. The size distribution of the liposomes was measured with a DLS Malvern Nano-ZS Zetasizer (Malvern Instruments Ltd.; Malvern, Worcestershire, UK). The Zetasizer was loaded with a liposome solution ratio 1 : 200 (70 *μ*M) in 1X phosphate-buffered saline (PBS, pH 7.4) solution and ran 13 cycles to obtain a measurement. This instrument is also used to obtain the zeta potential charge. DPPC liposomes have a resultant negative potential of −11.4 mV, indicating that the liposomes are stable and that they resist aggregation. Data were analyzed using the Dispersion Technology Software version 4.20 (Malvern Instruments Ltd.). 

### 2.4. Preparation of Long-Circulating Gold-Liposomes

Conjugated spherical gold nanoparticles with a diameter of 90 nm (Nanopartz) were covalently linked to the long-circulating liposomes for AFM detection. “Gold-liposomes” were prepared by attaching the gold nanoparticles at the distal terminals of DSPE-PEG (2000)-COOH linker lipids, which have free carboxylic groups available for activation [9, 13]. After the dry lipid hydration and liposome isolation procedures described above, 300 *μ*L of the 30 mM liposome solution was incubated with the activating reagents EDC (9 mg) and sulfo-NHS (11 mg) for 4 hrs at room temperature. The free activating reagents were then removed by dialysis through a 6,000–8,000 MW cut-off membrane against PBS (pH 7.4). Then, 100 *μ*L of gold nanoparticles was added to 400 *μ*L of the activated liposome solution and incubated overnight at room temperature. The unbounded gold nanoparticles were removed by dialysis through a 500,000 MW cut-off membrane against PBS (pH 7.4), and the average size of the “gold-liposomes” was measured by DLS.

### 2.5. Atomic Force Microscopy

#### 2.5.1. Liposome Imaging

The liposomes were deposited on mica for AFM analysis by using a modification of the procedure of Ramachandran et al. [14]. Ruby red mica circles (11 mm diameter) were first glued to a 3′′ × 1′′ glass slide with Scotch super glue gel. Both uncoupled liposomes and gold-coupled liposomes were left to stand on freshly cleaved mica during 10 min. The liposomes were then fixed with 10% neutral buffered formalin for 10 min, washed 3 times with ultrapure water (Barnstead water system, Thermo Scientific; Dubuque, IA, USA), and dried in a sterilGARD III hood flow before scanning. AFM liposome imaging was performed in tapping mode in air with RTESP cantilevers (*fo* = 262–325 kHz, *k* = 20–80 N/m).

#### 2.5.2. Cell Imaging

For the endocytosis studies, 2 × 10^4^ cells/well of HCAECs were seeded in a collagen-(50 *μ*g/mL) coated 8-well slide system, with EBM-2 supplemented with an EGM-2 Bulletkit (SingleQuots) to obtain 80% confluence after 24 hr of incubation at 37°C in a 5% CO_2_ atmosphere. The cells were then treated with the gold-labeled liposomes at a final concentration of 1.525 mM (solution ratio 1 : 10 in culture medium) for four different incubation times (15, 30, 60, and 120 min) at 37°C. During the incubations, the slide chambers were agitated at 30 rpm with an Orbit P4 Digital Shaker (Labnet, Edison, NJ, USA). The cells were then washed 3 times with culture medium to remove unbounded liposomes and fixed for 15 min with 10% neutral buffered formalin. 

AFM studies were performed on “never-dried” fixed cells, after the four incubation periods, to investigate liposome-membrane interactions. Liquid scanning was performed in contact mode with DNP-S cantilevers (*fo* = 12–24 kHz, *k* = 0.06 N/m). AFM was performed with a BioScope II Controller (Bruker Corporation). Image analysis was conducted with the Research NanoScope software version 7.30. For routine liposome detection, we used a Nikon TE2000-E inverted optical fluorescence microscope (Nikon Instruments, Inc. Lewisville, TX, USA) integrated to the bioscope system. Epifluorescence images were taken using an FITC-HYQ filter set (excitation 460–500 wavelengths) with 20 and 40X objectives, respectively. The cells that emitted a positive fluorescent signal by the FITC-labeled liposomes were selected for AFM scanning. The results from the liposome AFM measurements were also verified by DLS analysis.

### 2.6. Inhibition of Endocytosis

The effect of dynasore monohydrate on cellular uptake of liposomes was also investigated. Dynasore monohydrate is a GTPase inhibitor that targets dynamin and blocks endocytosis [15, 16]. Dynamin has an essential role of vesicle formation in receptor-mediated endocytosis [17, 18]. The dynasore monohydrate was initially diluted to 20 mM in DMSO (99.9%) and stored in 20-*μ*L aliquots at −20°C. At the time of the experiments, it was diluted to 80 *μ*M (0.4% DMSO) in EBM-2 that contained no serum or albumin [19]. The HCAECs were then treated with dynasore monohydrate (80 *μ*M) for 15 min and agitated at 30 prm at 37°C before being incubated for 60 min with gold-labeled liposomes diluted 1 : 10 (1.525 mM) in EBM-2 supplemented with the EGM-2 BulletKit. We chose to investigate dynasore monohydrate's blocking effect after 60 min of incubation because, as described later, we learned that the “gold liposomes” had been taken up by the cells at that point in time. This is also in agreement with the work of Mastrobattista et al. [20], who reported that the 60% of cell-bound immunoliposomes are taken up by bronchial epithelial cells within 1 h of incubation. 

## 3. Results and Discussion

Our initial attempts to monitor the endocytosis of FITC liposomes by HCAECs required the utilization of an imaging contrast agent to improve visual enhancement during AFM scanning. FITC-labeled liposomes were not detectable during AFM imaging when incubated with HCAECs (see Figure S1 in Supplementry Materials avalible online at http://dx.doi.org/10.1155/2013/875906). Positive fluorescence signaling was detected from the fluorescently labeled lipid, but uncoupled liposomes were not detectable on the surface of the cell membrane during AFM scanning. This difficulty led us to use colloidal gold to perform single-molecule tracking, a common technique practiced in morphological studies [7, 8]. For this reason, 90 nm gold particles were coupled to FITC-labeled liposomes to facilitate particle identification in AFM profiles. 

### 3.1. Characterization of Uncoupled Liposomes and Gold-Coupled Liposomes

#### 3.1.1. DLS Measurements

**Figure 1:**
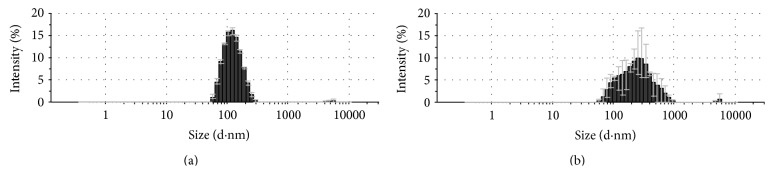
The hydrodynamic radii of noncoupled (a) and gold-coupled liposomes (b), estimated by DLS. Uncoupled liposomes (a) observed a mean diameter value of 129 ± 1.3 nm. The size distribution for gold-labeled liposomes (b) described a major peak for particles about 285 ± 5.3 nm in size. DLS results represent the averages of 13 runs at three different measurements per sample.

The average diameter of the non-gold-coupled liposomes, as determined by DLS, was 129 ± 1.3 nm (Figure 1(a)), and a polydispersity index (PDI) of 0.126. The DLS results represent the averages of three different measurements (13 runs each) per sample. For the gold-coupled liposomes (Figure 1(b)), the DLS results showed a major peak at 285 ± 5.3 nm. The PDI for the gold-liposomes was 0.689.

The polydispersity index indicates the variation in particle size. Its denomination can vary from 0 to 1. A value of 1 means that the sample is very polydisperse and a value of 0 means that the particle size does not vary. Values below 0.2 indicate that a sample is monodisperse [21, 22]. Therefore, the PDI values reported here indicate that the noncoupled liposomes were very monodisperse. However, the gold-coupled liposomes had a more heterogeneous formulation, some containing only one or two gold particles and some having many (clusters). 

#### 3.1.2. AFM Analysis of Liposomes

**Figure 2:**
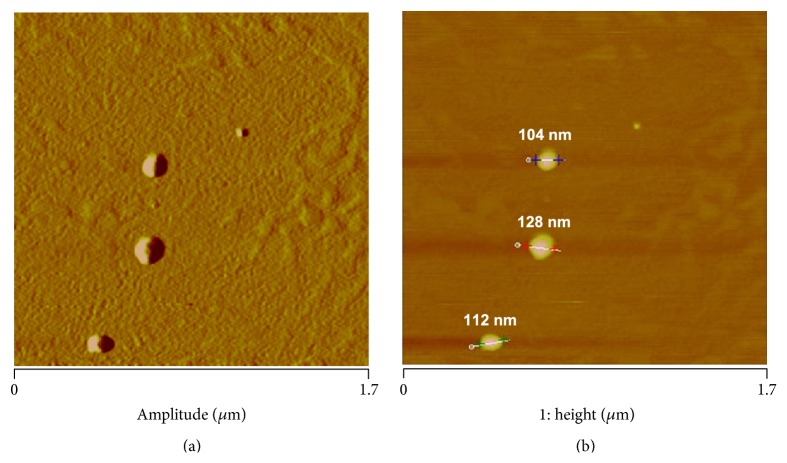
AFM images of non-gold-coupled liposomes visualized in amplitude (a) and height (b) modes to 1.7 *μ*m (*x*-*y*). The AFM section analysis of these liposomes showed an average diameter of 121.5 ± 27 nm when deposited on mica and scanned in air. This result was comparable to that of the DLS analysis (129 ± 1.3 nm). Scan obtained in tapping mode using RTESP tips (*fo* = 262–325 kHz, *k* = 20–80 N/m).

The structural properties of uncoupled and gold-coupled liposomes were analyzed using AFM. Both types of liposomes were imaged after fixation with 10% formalin to ensure the preservation of their original structure. AFM was performed in tapping mode in air with RTESP cantilevers.  Figure 2 shows a typical AFM image of non-gold-coupled liposomes deposited on fresh cleaved mica and fixed with formalin. AFM analysis of these liposomes showed an average diameter of 121.5 ± 27 nm. This result was comparable to the diameter measurement obtained by DLS (129 ± 1.3 nm). 

**Figure 3:**
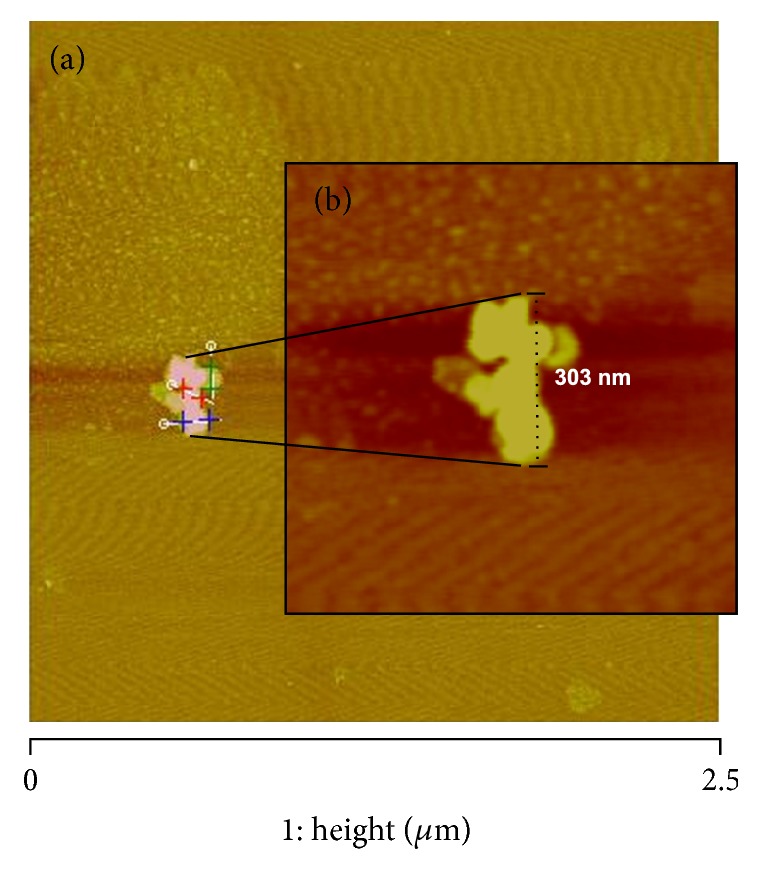
AFM images of FITC-labeled liposomes coupled to 90 nm gold particles and scanned on fresh cleaved mica. (a) Height image with section analysis measuring the diameter of three particles (95, 86, and 80 nm, resp.) that made up a typical gold-liposome cluster. (b) Additional digital zoom to 1 *μ*m^2^ taken from the original scan at 2.5 *μ*m (*x*-*y*). The DLS analysis showed that the gold-liposome complexes had a diameter of 285 ± 5.3 nm. Tapping mode in air using RTESP cantilevers (*fo* = 262–325 kHz, *k* = 20–80 N/m).

To enhance AFM imaging, nanogold particles were covalently linked to FITC liposomes. We choose 90 nm gold particles to facilitate visualization within the cell membrane taking into consideration that ~120 nm gold nanoparticles are normally used for thermal ablation.  Figure 3 shows AFM imaging of the geometric structure of a typical gold-liposome cluster (303 nm in size) consisting of three distinctive particles with individual diameters of 95, 86, and 80 nm.

According to DLS measurements, these gold-liposome complexes had an average size of 285 ± 5.3 nm (3 different measurements with 13 runs each). The arrangement and geometrical structure of the gold-liposome clusters changed, but the average size remained similar in different formulations (*n* = 5).

### 3.2. Endocytosis and Liposome Coupling

**Figure 4:**
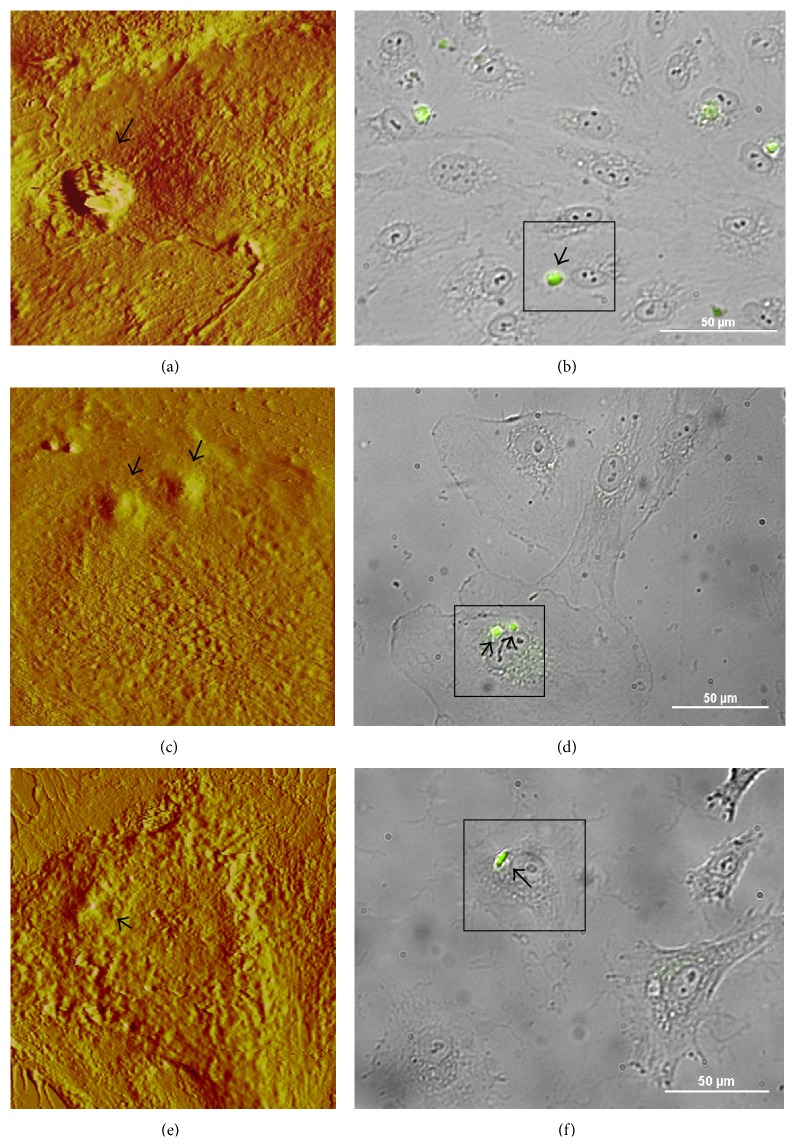
AFM images ((a), (c), and (e)) and corresponding fluorescence images ((b), (d), and (f)) of HCAECs incubated for 15 ((a) and (b)), 60 ((c) and (d)), and 120 min ((e) and (f)) with FITC-labeled liposomes conjugated with 90 nm gold particles. The cells with positive signaling in the bright field images (squares in the right-hand panels) were selected for AFM scanning. (a) Typical AFM image of an HCAEC incubated for 15 min, showing a gold liposome cluster attached to the cell membrane. (c) AFM image demonstrating the internalization process occurring in an HCAEC incubated for 60 min with “gold liposomes.” (e) The AFM micrograph of an HCAEC incubated for 120 min corroborated that the “gold liposomes” internalized almost completely at this time point. Cells fixed in formalin 10% and scanned in contact mode in liquid (DNP-S *fo* = 12–24 kHz, *k* = 0.06 N/m).

The surface topology and characteristics of biological membranes can routinely be described using biological AFM instruments [23, 24]. Nevertheless, the application of this technique has rarely been approached to resolve kinetics for liposome cell uptake. To investigate how endocytosis takes place within endothelial cells, we used 90 nm gold particles to track FITC-labeled liposomes.  Figure 4 shows sequential AFM images illustrating how the “gold-liposomes” internalize the plasma membrane. 

**Figure 5:**
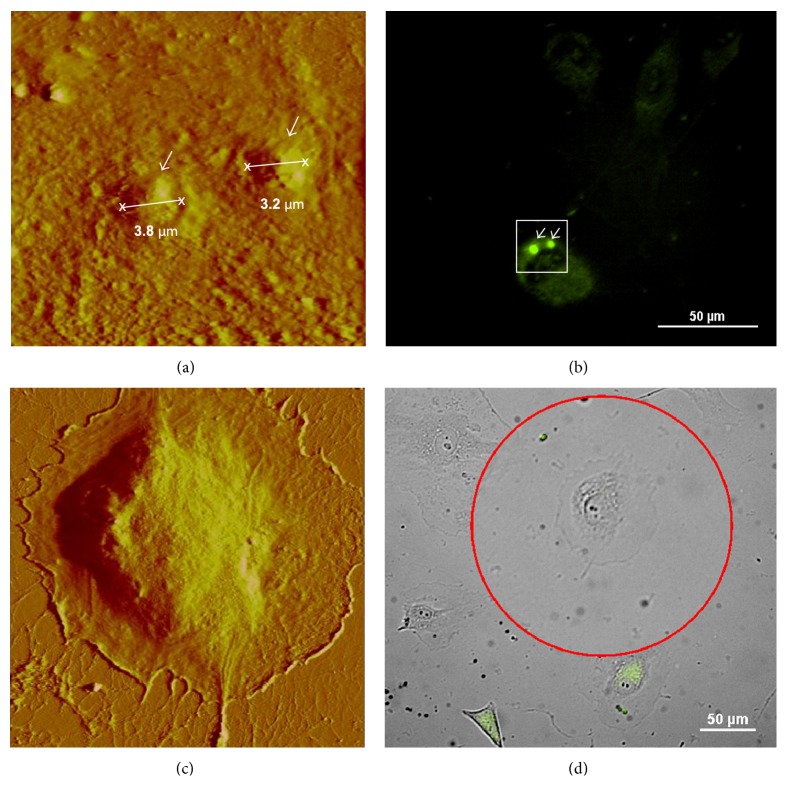
Liposome endocytosis occurring at 60 min of incubation (smaller scan area from Figure 4(c)). (a) AFM micrograph obtained at 25 *μ*m (*x*-*y*) illustrating the engulfment of the “gold liposomes” during cell membrane internalization. (b) The corresponding fluorescence image clearly shows the positive signaling emitted by the gold liposome clusters that were scanned. (c) AFM micrograph at 65 *μ*m (*x*-*y*) of an HCAEC negative control showing a smooth and even membrane surface after 60 min of incubation with FITC-labeled gold-liposomes. (d) The cells with no signalling, which indicated the absence of “gold-liposomes,” were selected for scanning from the corresponding fluorescence images. Cells were fixed in formalin 10% and scanned in contact mode in liquid (DNP-S *fo* = 12–24 kHz, *k* = 0.06 N/m).

After 15 min of incubation (Figures 4(a) and 4(b)), some of the coupled liposomes were already attached to the cells. They still quivered when probed with the AFM tip during scanning (seen as liposome drifting in Figure 4(a)). Nevertheless, the “gold-liposomes” were strongly bonded to the cell membrane at this time, given that the liposomes remained attached to the cell when probed. At 30 min, the liposome clusters started to enter the cells (not shown). Actual endocytosis was seen in samples incubated for 60 min (Figures 4(c) and 4(d)). Figures 4(c) and 4(d) clearly show how the plasma membrane is enclosing the extracellular material and gradually engulfing it (see also Figure 5(a), at a smaller scan area). The corresponding fluorescence images in Figures 4(d) and 5(b) show strong positive signals of the two “gold-liposome” clusters (indicated by arrows) that were selected for AFM scanning. One of these clusters was about 3.2 *μ*m and the other was 3.8 *μ*m in diameter. Liposomes incubated with HCAECs for 120 min were almost completely internalized (Figure 4(e)). 

These studies demonstrated that using colloidal gold nanoparticles as an image-enhancement tool did not hinder endocytosis and that uptake of the liposomes took place in 120 min. The time course of the endocytosis can be summarized as follows: liposome attachment to the plasma membrane (15–30 min), internalization (30–60 min), and liposome uptake completed (120 min). These results agree with the findings reported by Ramachandran et al. [14], who studied endocytosis of cisplatin-encapsulated liposomes. In that study, the cisplatin produced liposomes significantly stiffer than nonencapsulated liposomes, which facilitated their detection by AFM scanning.

On the other hand, our negative controls (HCAEC incubated with “gold-liposomes” for 60 and 120 min, which did not show signals detectable by fluorescence imaging) had smooth and even membrane surfaces (Figures 5(c) and 5(d)). Signs of elevated or raised areas were not present during AFM scanning, and the lack of fluorescence signaling indicated the absence of FITC-labeled gold-liposomes. 

### 3.3. Blocking Gold-Liposome Uptake

**Figure 6:**
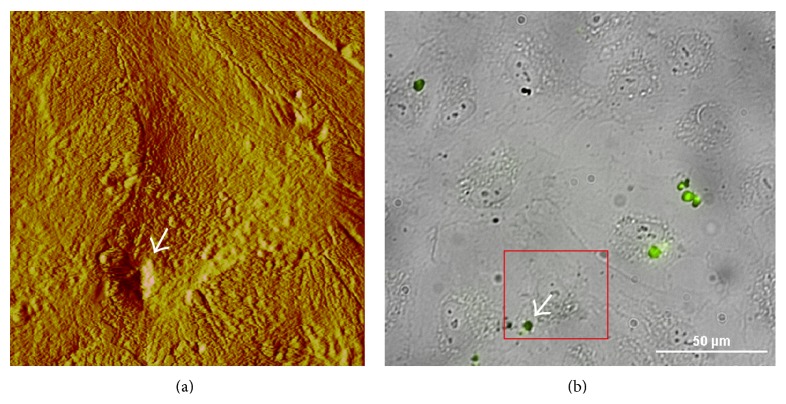
The internalization process of coupled liposomes was blocked when the cells were pretreated with 80 *μ*M dynasore for 15 min before 60 min of incubation with “gold-liposomes.” The gold liposome clusters did not penetrate the cell membrane, remaining just attached to the external wall (arrow in (a)). The corresponding fluorescence image (b) shows positive signaling from the “gold liposomes” that were scanned. The cells were fixed in formalin 10% and imaged at 50 *μ*m^2^ in contact mode in liquid (DNP-S *fo* = 12–24 kHz, *k* = 0.06 N/m).

Endocytosis is the process that cells use to take up nanovectors and other materials from the external environment, by encapsulating them in vesicles made from invaginations in the cell plasma membrane. It was of particular interest to investigate if the internalization of the gold-coupled liposomes could be hindered by a typical inhibitor of endocytosis. We used dynasore, a cell permeable, noncompetitive dynamin GTPase activity inhibitor, to block dynamin-dependent endocytosis of the liposomes (Figure 6). Our experiments indicated that the “gold-liposome” clusters barely attached to the external walls of cells treated 15 min with 80 *μ*M dynasore and then incubated with the “gold-liposomes” for 60 min. In most of the cases, the “gold-liposomes” were easily removed from the cell membrane when probed with the AFM cantilever. The force loading exercised by the AFM probe, scanning to a velocity of 30 *μ*m/s, removed ~90% of the liposomes. This indicated a poor binding efficiency with the plasma membrane and obstruction of the “gold-liposome” uptake after dynasore pretreatments. 

## 4. Conclusions

Ninety-nanometer colloidal gold particles are a useful noninvasive labeling agent for visualization by AFM of liposome uptake by HCAECs. We were able to visualize the movement of the gold-coupled liposomes through the cell membrane before absorption. The time course of endocytosis was as follows: liposome attachment to the plasma membrane at 15–30 min, internalization at 30–60 min, and liposome uptake completed at 120 min. “Gold-liposome” clusters up to ~3 *μ*m in diameter can be efficiently taken up by endocytosis regardless of their geometric structure. 

The gold-coupled liposomes behaved as expected when exposed to an endocytosis inhibitor (dynasore) to block their internalization process. The gold nanoparticles did not hinder liposome uptake. We successfully established a potential method to track biomolecules in complex systems using 90 nm colloidal gold nanoparticles as a noninvasive contrast agent to improve AFM imaging.

## Supplementary Material

AFM images of HCAECs incubated with FITC-labeled liposomes demonstrated that uncoupled liposomes were not detectable to the AFM technique as presented in the Figure S1. We provide supplementary information to support judgment on the use of colloidal gold nanoparticles as a noninvasive contrast agent to improve AFM imaging.
