# Supplementary material for: Biological Atomic Force Microscopy for Imaging Gold-Labeled Liposomes on Human Coronary Artery Endothelial Cells
Source: J Pharm (Cairo). 2013 Feb 21;2013:875906. doi: 10.1155/2013/875906 (PMC4590807; doi:10.1155/2013/875906)
Supplement: Supplementary file 1 — AFM images of HCAECs incubated with FITC-labeled liposomes demonstrated that uncoupled liposomes were not detectable to the AFM technique as presented in the Figure S1. We provide supplementary information to support judgment on the use of colloidal gold nanoparticles as a noninvasive contrast agent to improve AFM imaging. [file 875906.f1.pdf]

Supplementary Data

Using Biological Atomic Force Microscopy to Image Gold-Labeled Liposomes at Human Coronary Artery Endothelial Cell Membranes

Ana-María Zaske [a,], Delia Danila [a], Michael C. Queen [a], Eva Golunski [b], Jodie L. Conyers [c,1]*

Division of Cardiology, Department of Internal Medicine, The University of Texas Health Science Center at Houston Medical School

[a] *1881 East Road, Houston, TX 77054, USA*

[b] *1841 East Road, Houston, TX 77054, USA*

[c] *6410 Fannin Street, Houston, TX 77030, USA*

[*]To whom correspondence should be addressed: Email: ana.m.zaske@uth.tmc.edu

Experiments conducted on control cells (uncoupled liposomes).

The internalization process of standard liposomes (FITC-labeled liposomes) was investigated in HCAECs using the AFM technique. The results of preliminary experiments demonstrated that FITC-liposomes (uncoupled liposomes) were not visible to the AFM technique. The Figure S1 is a typical representation of the cell topography after incubation with FITC-liposomes. We observed that the utilization of a contrast agent (colloidal gold particles) efficiently improved the detection of liposomes within the cell membrane. In this paper we describe a potential method to track biomolecules in complex systems using 90 nm colloidal gold nano-particles to resolve AFM imaging.

[1] Present address: 1901 Pennsylvania Ave NW, Washington, DC 20006, USA

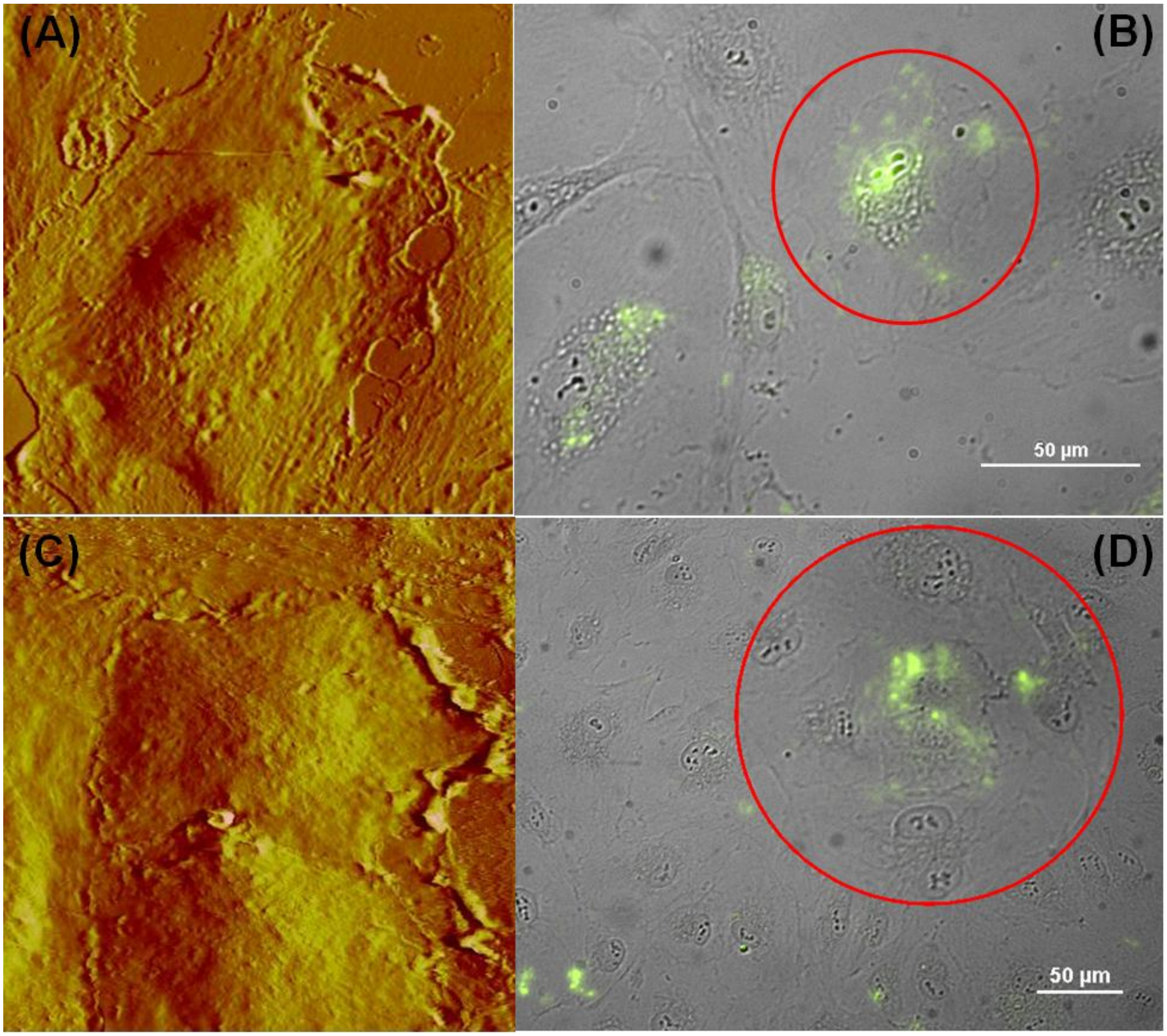

Figure S1. HCAECs incubated with FITC labeled liposomes (non-gold coupled) for 30 min (A and B) and 60 min (C and D). The cells with positive signaling in the bright-field images (circles in the right-hand panels) were selected for AFM scanning. The fluorescence images (B and D) showed that the FITC liposomes were randomly dispersed on the cell membrane. AFM imaging (A and C) demonstrated smooth membrane surfaces. FITC-liposomes were not visualized on HCAECs treated with uncoupled liposomes for 30 (A) and 60 min (C). Cells fixed in formalin 10% and scanned at 70 (A) and 60 µm^2 (C) in contact mode in liquid (DNP-S f_o=12-24 kHz, k=0.06 N/m).
